# Supplementary material for: Unlocking carrier confluence in covalent organic frameworks for efficient photoreduction of dilute nitrate to ammonia
Source: Nat Commun. 2026 Feb 24;17:3141. doi: 10.1038/s41467-026-69439-4 (PMC13044306; doi:10.1038/s41467-026-69439-4)
Supplement: Supplementary file 1 — Supplementary Information [file 41467_2026_69439_MOESM1_ESM.pdf]

Supplementary Information

Unlocking carrier confluence in covalent organic frameworks for efficient photoreduction of dilute nitrate to ammonia

Yang Su[1,2], Zhe Wang[1], Xiaoxu Deng[2,*], Shuang-Feng Yin[3,*], and Peng Chen[1,2*]

[1]Guizhou Provincial Key Laboratory of Green Catalysis and Materials for Resource Conversion, School of Chemistry and Chemical Engineering, Guizhou University, Guiyang 550025, Guizhou, China.

[2]College of Big Data and Information Engineering, Guizhou University, Guiyang 550025, Guizhou, China

[3]College of Chemistry and Chemical Engineering, Hunan University of Science and Technology, Xiangtan 411201, P. R. China; Advanced Catalytic Engineering Research Center of the Ministry of Education, State Key Laboratory of Chemo/Biosensing and Chemometrics, College of Chemistry and Chemical Engineering, Hunan University, Changsha 410082, P. R. China.

* Correspondence: dxjdeng7@163.com (X. Deng), sf_yin@hnu.edu.cn (S.-F. Yin), pchen3@gzu.edu.cn (P. Chen)

Supplementary Tables

Supplementary Table 1. Calculated dipole moments for PI and PIS.

	**PI**	**PIS**
μ_g	6.5553	7.9391
μ_e	6.5974	7.9776

Supplementary Table 2. Zeta potentials of as-prepared photocatalysts.

**Samples**	**1**	**2**	**3**	**4**	**5**	**average**
PI	-3.88	-3.35	-3.71	-3.89	-3.54	-3.67
PIS	-11.16	-9.49	-9.34	-10.25	-10.49	-10.15

Supplementary Table 3. The effective mass (m*) and SE coupling of electrons and holes.

**Sample**	**E (eV)**	**E (eV)**	**V^{eff} (eV)**	**a(Å)**	**m*(h^+)**	**m*(e^-)**
PI	E_{HOMO}^{DAD}	E_{HOMO-1}^{DAD}	$V_{h^+}^{eff}$			
	-5.158	-5.162	0.002	1.412	7.833E-07	
	E_{LUMO}^{ADA}	E_{LUMO+1}^{ADA}	$V_{e^-}^{eff}$			
	-4.347	-4.298	0.024	1.408		7.054E-08
PIS	E_{HOMO}^{DAD}	E_{HOMO-1}^{DAD}	$V_{h^+}^{eff}$			
	-5.718	-5.961	0.121	1.407	1.422E-08	
	E_{LUMO}^{ADA}	E_{LUMO+1}^{ADA}	$V_{e^-}^{eff}$			
	-3.628	-2.557	0.535	1.408		3.222E-09

The m* of electrons-holes serves as an effective means to evaluate charge transport rates and polarity. For donor-acceptor (D-A) model organic copolymers, the m* depends on the super-exchange mechanism between D and A.

The formula for calculating V^{eff} is as follows:

$$V_{h^+}^{eff}=(E_{HOMO}^{DAD}-E_{HOMO-1}^{DAD})/2$$

$$V_{e^-}^{eff}=(E_{LUMO+1}^{ADA}-E_{LUMO}^{ADA})/2$$

The m* can be calculated by the following equation:

$$m^*=-\frac{h^2}{2\mathbf{a}^2 V^{eff}}$$

Where h, a represent Planck's constant, the distance between adjacent D and A is one-half of the size of the unit cell (a). Here, the plus or minus sign represents direction, therefore, for comparison, the m* of electrons and holes are both taken as positive values[1].

Supplementary Table 4. Time-resolved fluorescence decay parameters of all samples.

Sample	A_1 (%)	τ_1 (ns)	A_2 (%)	τ_2 (ns)	τ (ns)
PI	95.90	0.1855	4.10	7.1092	4.485
PIS	95.06	0.1881	4.94	7.2255	4.877

The I_t and τ can be calculated by the following equation:

$$I_t = A_1 * \exp\frac{-t}{\tau_1} + A_2 * \exp\frac{-t}{\tau_2}$$

$$\tau = \frac{(A_1\tau_1^2 + A_2\tau_2^2)}{(A_1\tau_1 + A_2\tau_2)}$$

Supplementary Table 5. KIE values and activation free-energy differences (298 K).

Sample	NH_4^+ rate in H_2O (mmol g^{-1} h^{-1})	NH_4^+ rate in D_2O (mmol g^{-1} h^{-1})	KIE (k_H/k_D)	$\Delta G^{\#}$ (kJ mol^{-1})
PI	0.092	0.064	1.430	0.880
PIS	0.758	0.653	1.160	0.430

D_2O rates are back-calculated from measured $KIE=(rate_{H_2O}/rate_{D_2O})$.

$\Delta G^{\#}=RT\ln(KIE)$, where R = 8.314 J·mol^{-1} K^{-1}, T = 298 K.

To verify its enhancement effect on proton hydrogen diffusion, we performed H_2O/D_2O experiments, replacing H_2O with D_2O as the electrolyte solvent, to measure the kinetic isotope effect (KIE) and delve into the *H transfer kinetics during the nitrate reduction reaction (NO_3RR). The KIE values for PI and PIS, typically calculated as the ratios of ammonia yield/current density in H_2O to those in D_2O, are 1.43 and 1.16, respectively. All values above 1 reveal that H_2O dissociation and its role in hydrogenating intermediate nitrogen species are the rate-determining steps (RDS) across all samples[2]. In D_2O, all catalysts show a minor drop in NH_4^+ yield. Under identical conditions, PIS has the lowest KIE value, suggesting that the S=O=S group optimizes the catalytic interface, boosting *H transfer[3]. Conversely, PI has the highest KIE value, pointing to kinetic limitations in *H migration from water dissociation. The calculated activation free-energy differences ($\Delta G^{\#}=RT\ln(KIE)$) reinforce this: PI's $\Delta G^{\#}$ is 0.88 kJ·mol^{-1}, almost double PIS's (0.43 kJ·mol^{-1}). PIS's lower KIE and smaller $\Delta G^{\#}$

imply less reliance on an intact hydrogen-bonding network for proton transfer, aligning with the hypothesis of disrupted or weakened interfacial hydrogen bonds. This reduced proton-transfer sensitivity accounts for PIS's notably higher ammonia production and supports the idea that modifying the interfacial hydrogen-bonding environment enhances nitrate reduction efficiency.

Supplementary Figures

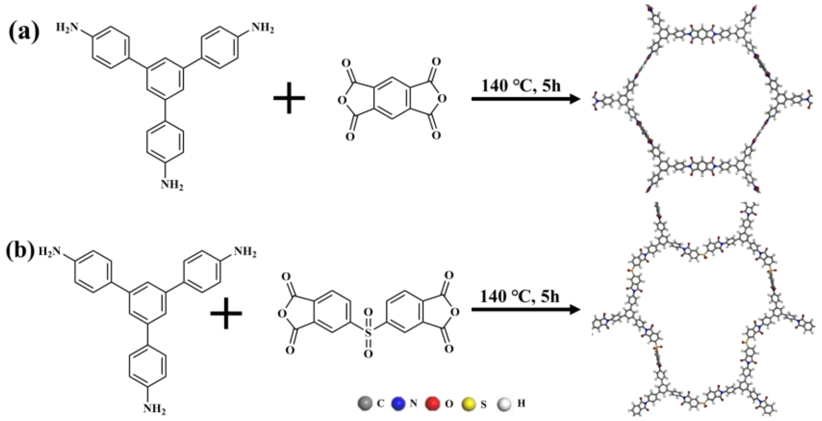

Supplementary Fig. 1. Synthetic routes of (a) PI and (b) PIS.

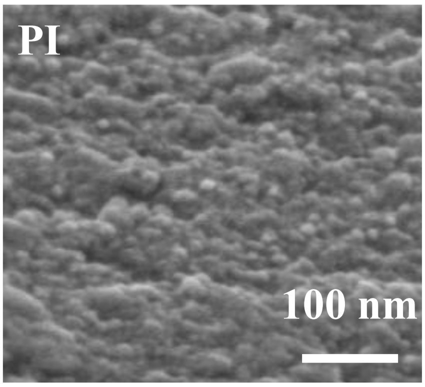

Supplementary Fig. 2. SEM image of PI.

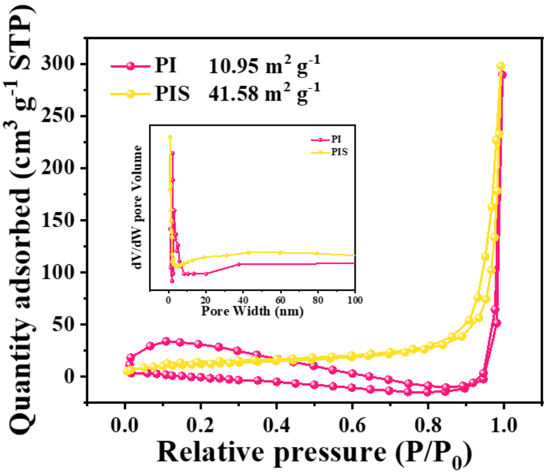

Supplementary Fig. 3. N_2 adsorption-desorption isotherms and pore size distributions of PI and PIS.

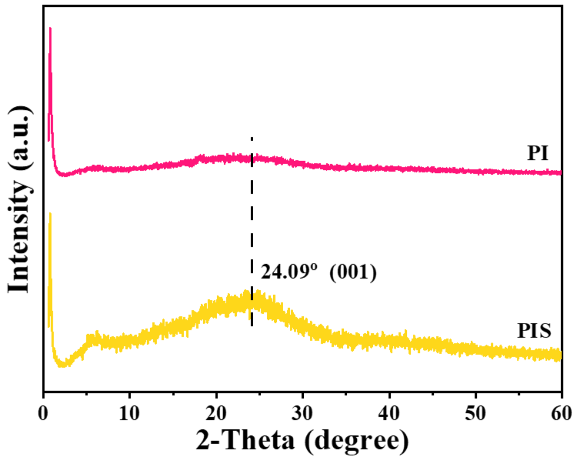

Supplementary Fig. 4. XRD patterns of as-prepared samples.

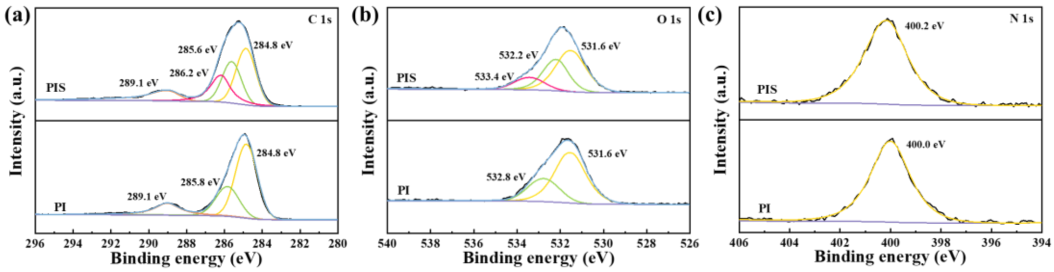

Supplementary Fig. 5. XPS spectra of (a) C 1*s*, (b) O 1*s* and (c) N 1*s* for PI and PIS.

C 1*s* XPS spectra showed that PIS exhibited deconvolution peaks at 284.8 eV (C=C), 285.6 eV (C-N), 286.2 eV (C-S), and 289.1 eV (C=O). The O 1*s* spectra of PIS at 531.6 eV (N-C=O), 533.4 eV (S=O), and 532.2 eV (surface adsorbed oxygen) each had one peak. Notably, the binding energy of C 1*s* in PIS shifted to lower values compared with that in PI. In the N 1*s* spectrum, PIS have a peak at 400.2 eV (N-C=O), while PI has a peak at 400.0 eV.

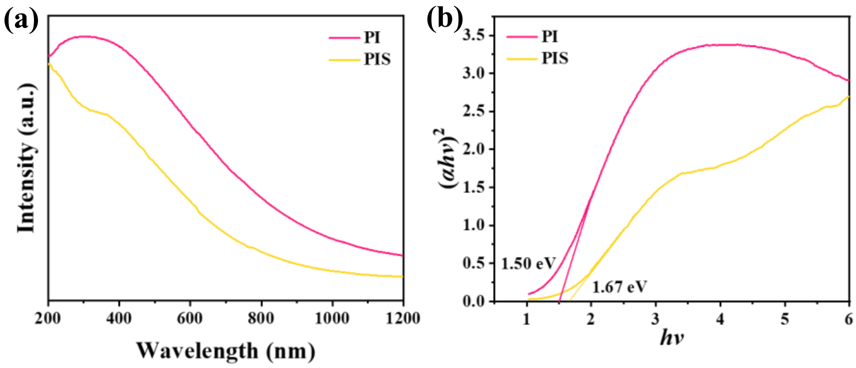

Supplementary Fig. 6. (a) UV-*vis* absorption spectra and (b) the corresponding Tauc plots of the fabricated samples.

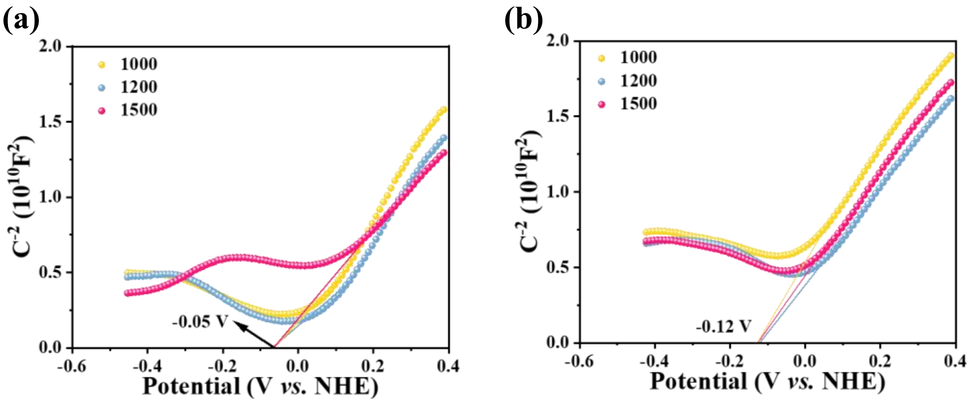

Supplementary Fig. 7. Mott-Schottky plots of fabricated samples: (a) PI and (b) PIS.

It is generally accepted that the flat band potential is approximately equal to the Fermi energy level and that for n-type semiconductors, the conduction band position is approximately 0.2 V below the Fermi energy level[4]. Therefore, the conduction bands of PI and PIS are -0.25 and -0.32 V, respectively.

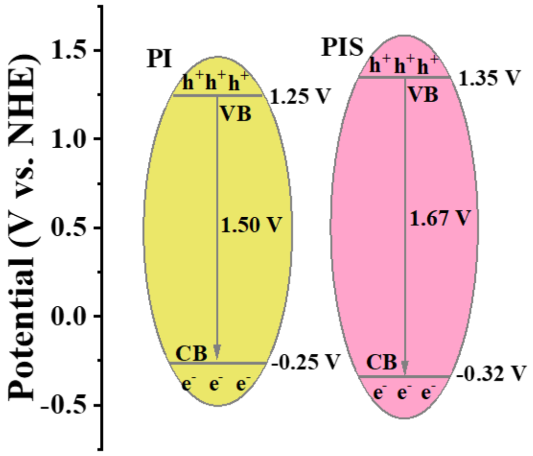

Supplementary Fig. 8. Schematic diagram of the electronic band structure of fabricated samples.

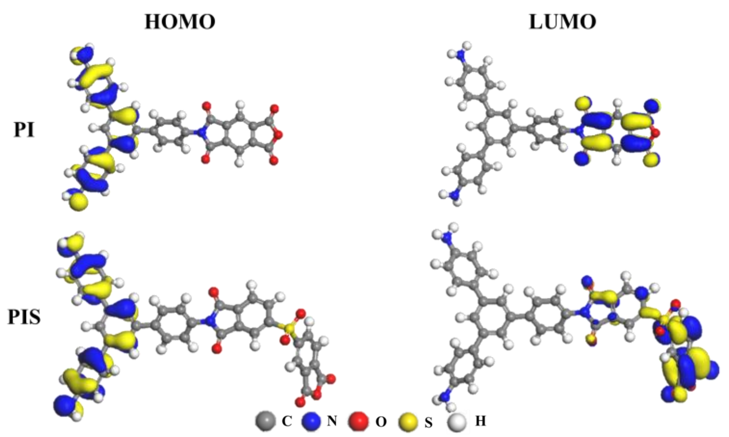

Supplementary Fig. 9. Frontier electron densities of HOMO and LUMO orbitals for PI and PIS.

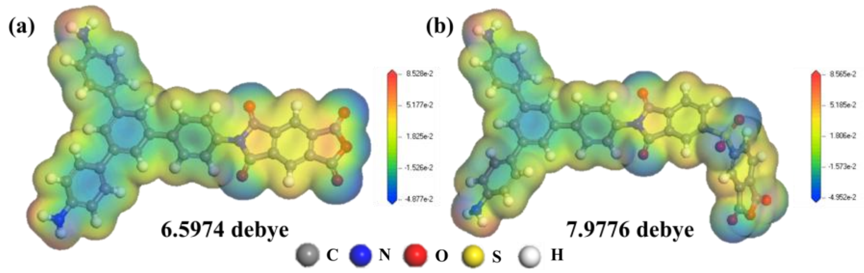

Supplementary Fig. 10. Frontier electron densities of surface electrostatic potential and molecular dipole for (a) PI and (b) PIS.

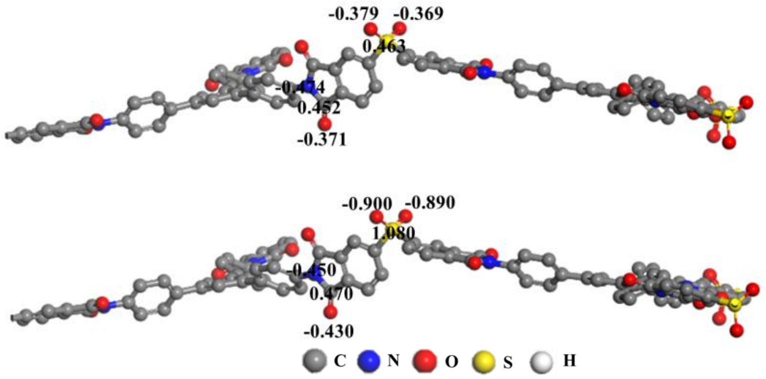

Supplementary Fig. 11. Frontier electron densities of *Mulliken* charge distribution on the bilayer PIS.

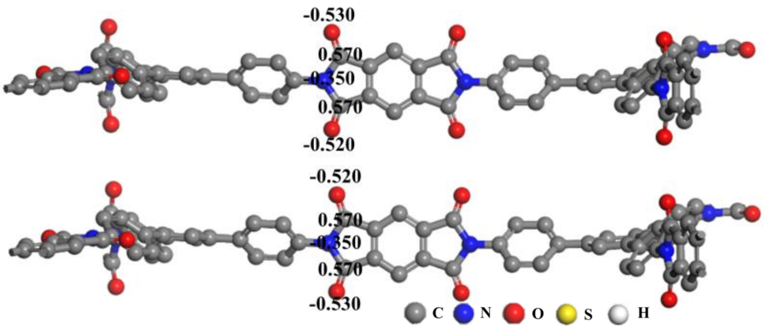

Supplementary Fig. 12. Frontier electron densities of *Mulliken* charge distribution on the bilayer PI.

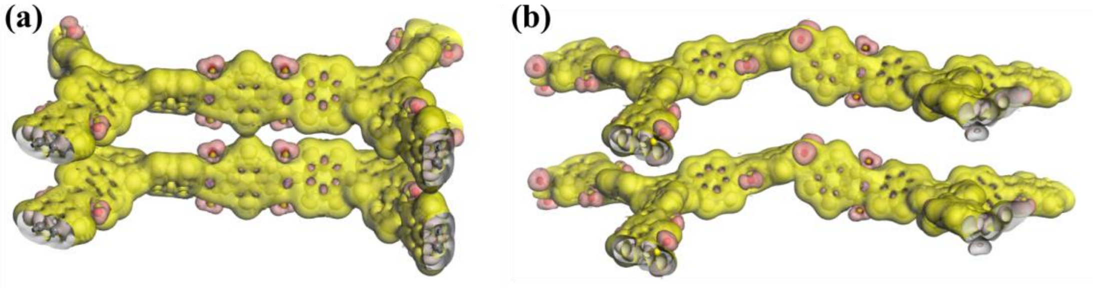

Supplementary Fig. 13. Theoretically calculated difference charge density distributions for (a) PI and (b) PIS.

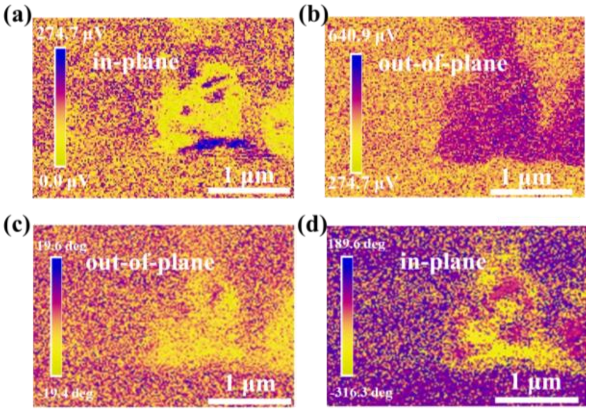

Supplementary Fig. 14. Piezoresponse force microscopy (PFM) of PI: (a) In-plane amplitude image, (b) out-of-plane amplitude image, (c) out-of-plane phase image and (d) in-plane phase image.

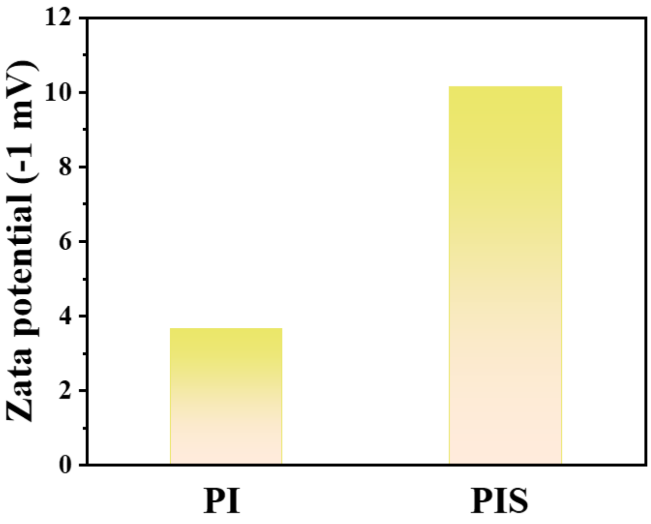

Supplementary Fig. 15. Zeta potential of PI and PIS.

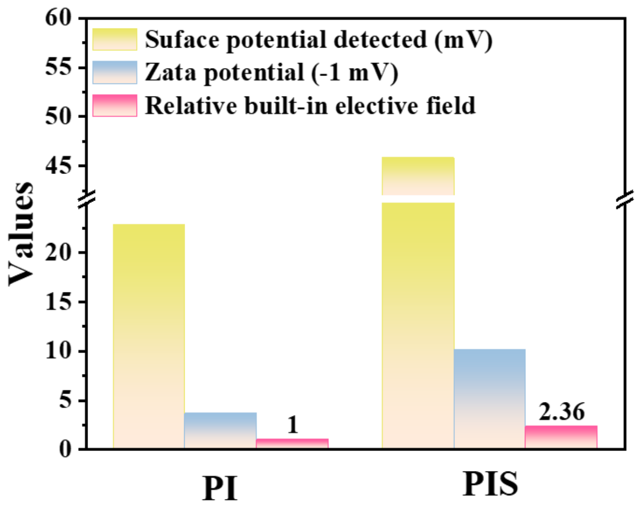

Supplementary Fig. 16. The relatively built-in electric field of as-prepared samples.

The specific calculation method of built-in electric field (BE) is as follows.

$$Fs=(-\frac{2Vs\rho}{\varepsilon\varepsilon_0})^{1/2}$$

Where Fs is the internal electric field magnitude, V_s is the surface voltage, ρ is the surface charge density, ε is the low-frequency dielectric constant, and ε_0 is the permittivity of free space. The above equation reveals that the internal electric field magnitude is mainly determined by the surface voltage and the charge density because ε and ε_0 are two constants[5, 6].

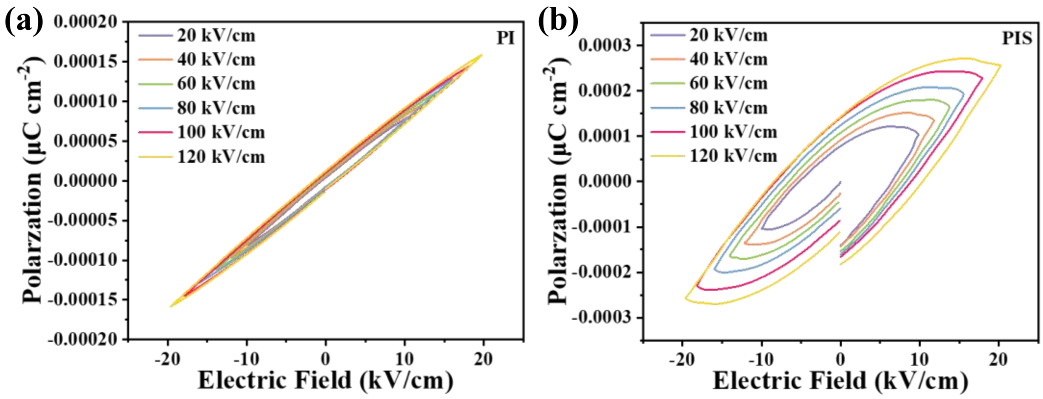

Supplementary Fig. 17. Room temperature hysteresis loops of (a) PI and (b) PIS.

Test process:

First, 0.1 g of the target sample was dissolved in 5 mL of distilled water and subjected to continuous ultrasound. The tea polyphenol solution was added half an hour before the preparation and the ultrasound was continued for half an hour. Then spin-coated on the 1×2 cm FTO conductive sheet with a 500 r/s coating instrument and dried. Then the 0.5 mm×0.5 mm electrode was sputtered by a small ion sputtering instrument. The ferroelectric curves under different field strengths were input into the ferroelectric test analyzer.

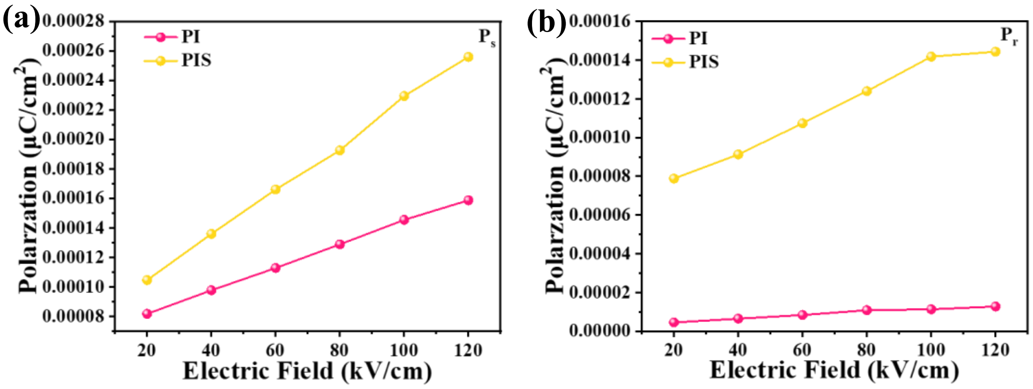

Supplementary Fig. 18. Room temperature hysteresis loop of (a) P_s and (b) P_r.

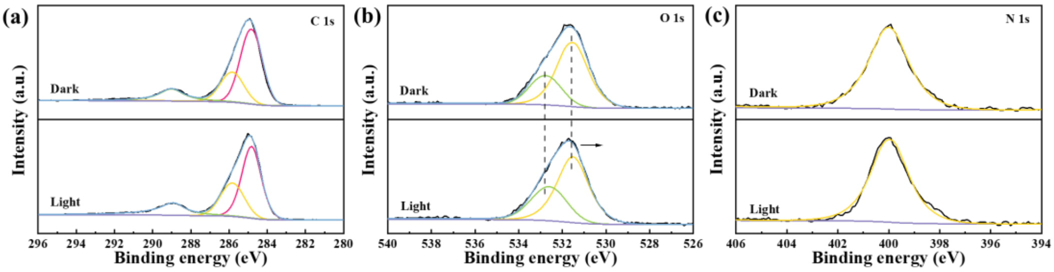

Supplementary Fig. 19. High-resolution *in-situ* XPS of PI: (a) C 1*s*, (b) O 1*s* and (c) N 1*s*.

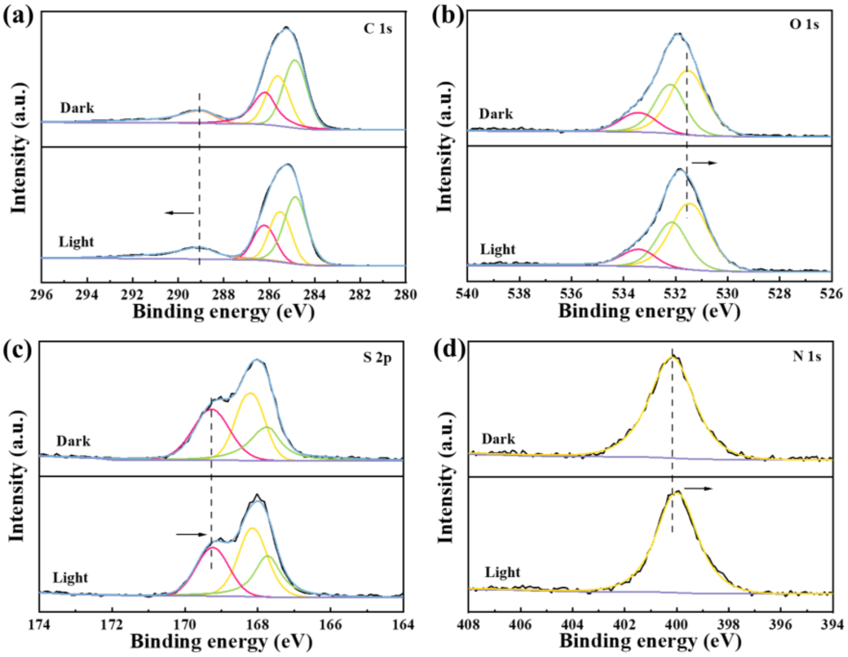

Supplementary Fig. 20. High-resolution *in-situ* XPS of PIS: (a) C 1*s*, (b) O 1*s*, (c) S 2*p* and (d) N 1*s*.

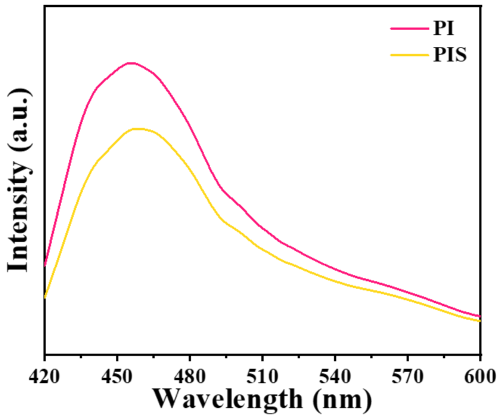

Supplementary Fig. 21. Photoluminescence spectroscopy of as-prepared samples.

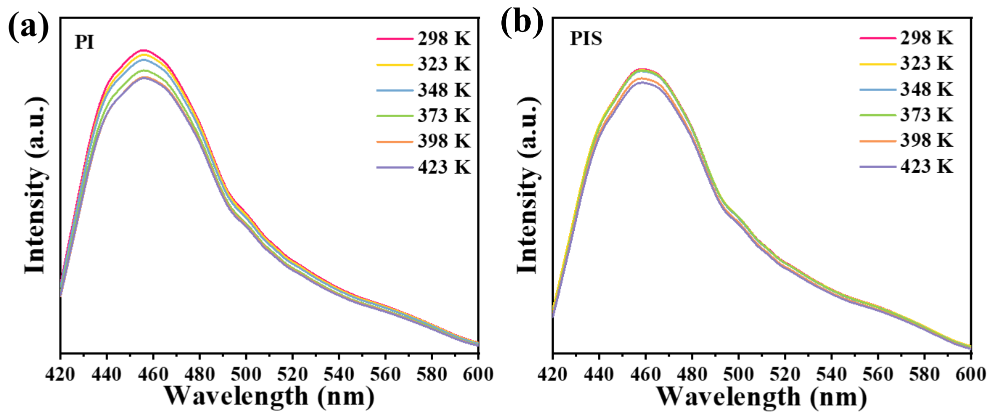

Supplementary Fig. 22. Temperature-dependent PL spectra of (a) PI and (b) PIS.

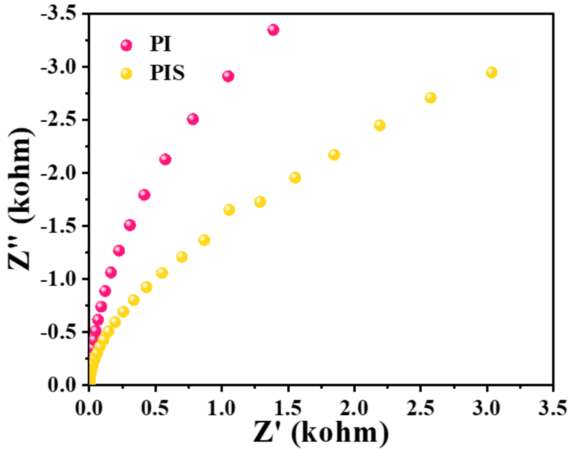

Supplementary Fig. 23. Electrochemical impedance spectroscopy of PI and PIS.

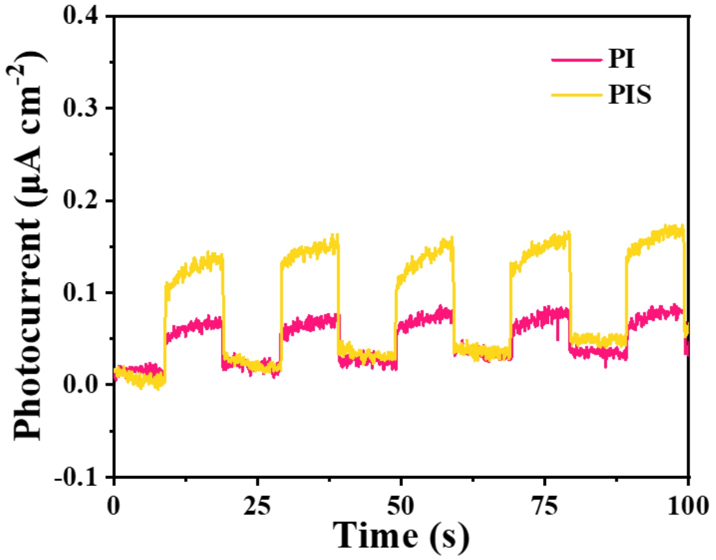

Supplementary Fig. 24. Photocurrent response spectra of the as-prepared samples.

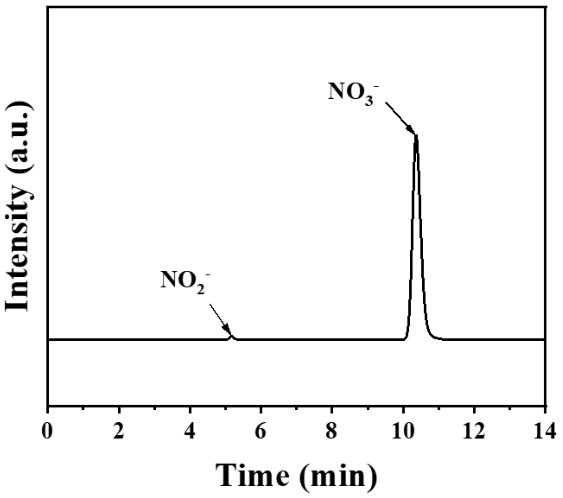

Supplementary Fig. 25. Ion chromatography is used to prove the product.

We extracted 1 mL of the reaction solution for detection by ion chromatography. The results showed that 1.54 × 10^{-3} mmol nitrite was produced during nitrate reduction.

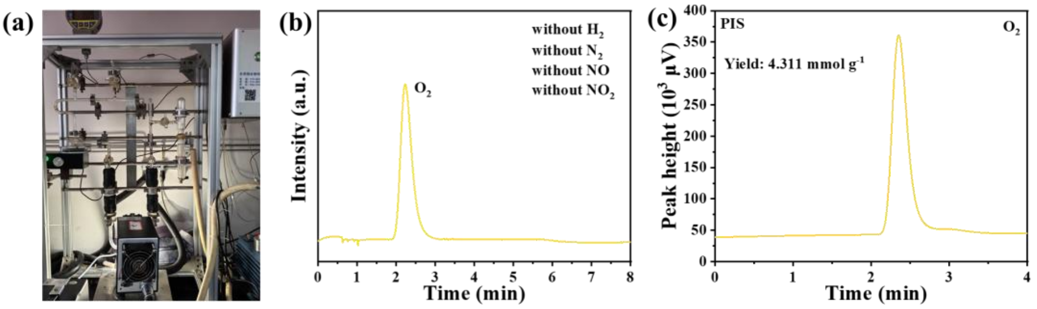

Supplementary Fig. 26. (a) Schematic image of the oxygen production experimental setup. (b) Gas chromatogram for the photocatalytic reduction of nitrate. (c) Quantitative experiment on photocatalytic reduction of nitric acid to ammonia.

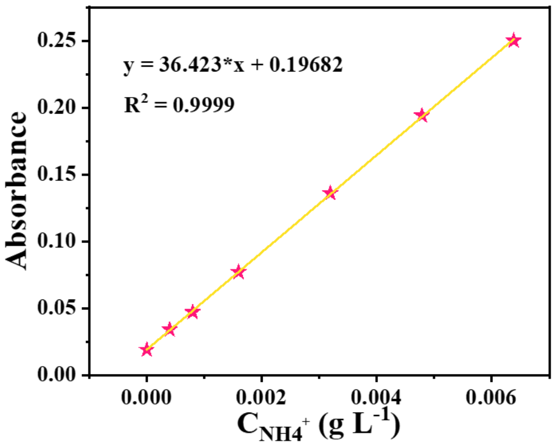

Supplementary Fig. 27. Calibration curves are used for absorbance of UV-*vis* curves at 420 nm to estimate NH_4^+ ion concentration.

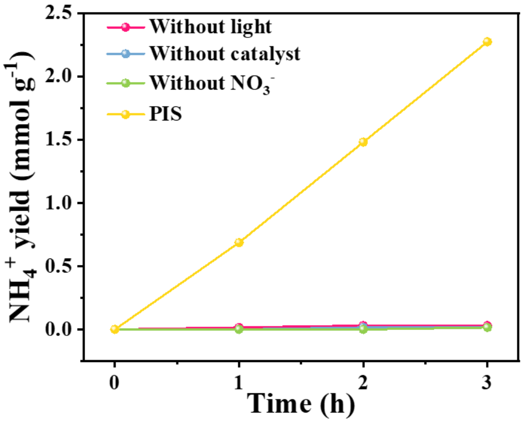

Supplementary Fig. 28. Comparison of NH_4^+ yield under different reaction conditions.

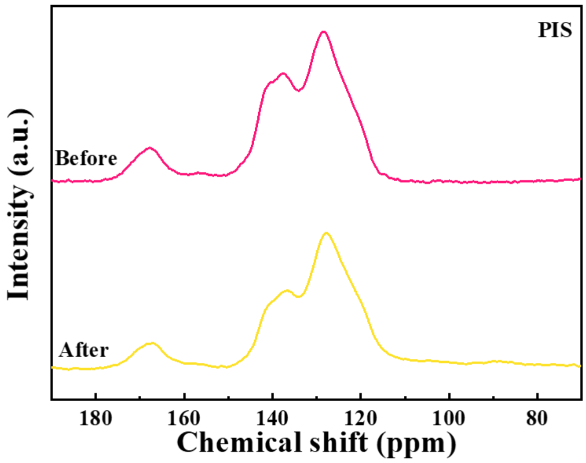

Supplementary Fig. 29. The solid-state ^{13}C NMR of PIS before and after the recycling test.

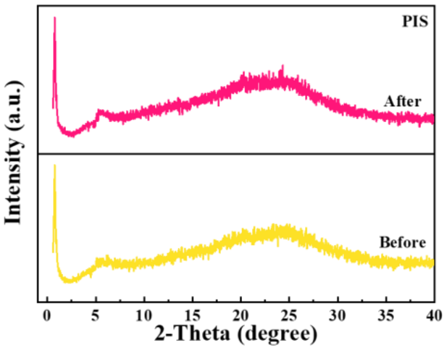

Supplementary Fig. 30. The XRD pattern of PIS before and after the recycling test.

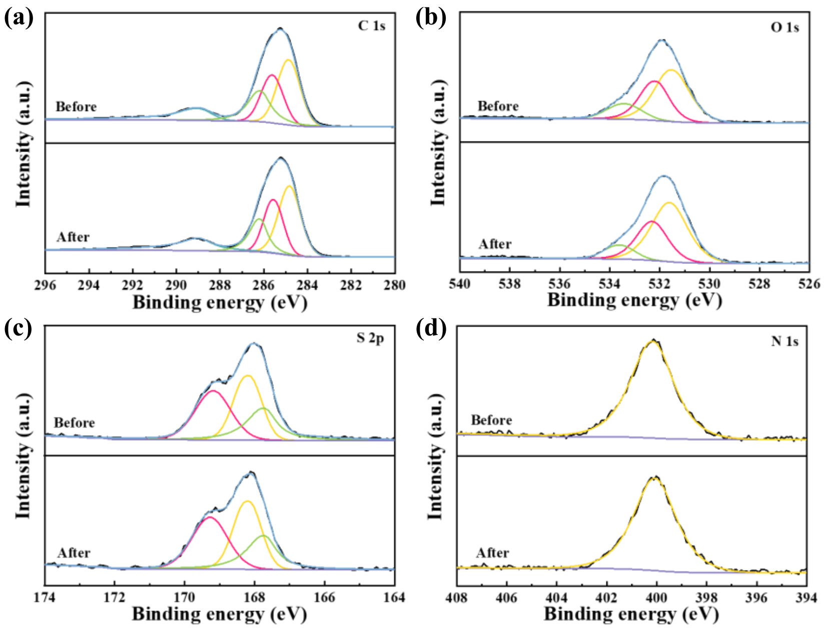

Supplementary Fig. 31. The XPS spectra of (a) C 1*s*, (b) O 1*s*, (c) S 2*p* and (d) N 1*s* for PIS before and after the recycling test.

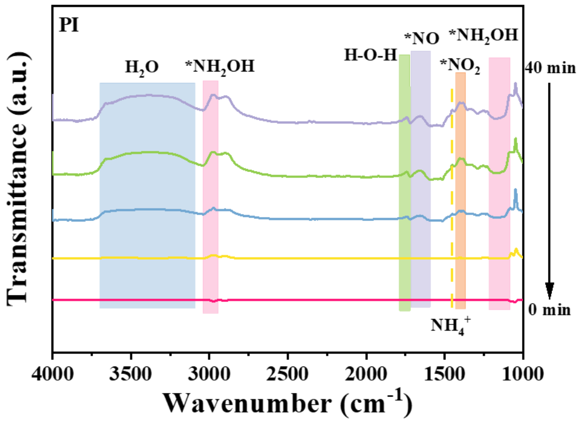

Supplementary Fig. 32. *In-situ* FTIR spectrum obtained from PI under NO_3^- solution conditions at different times.

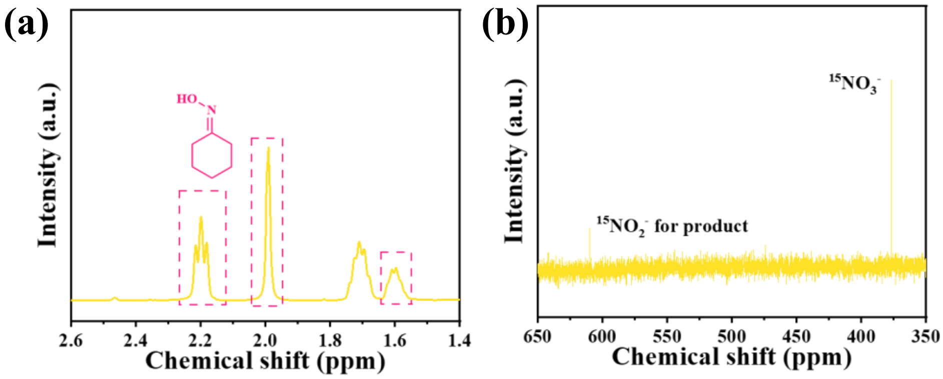

Supplementary Fig. 33. (a) ^{1}H NMR spectrum of the reaction product. (b) The reaction

product was detected by ^{15}N NMR spectra of PIS.

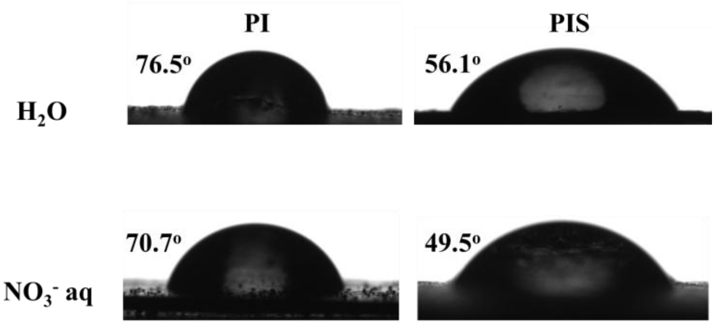

Supplementary Fig. 34. Contact-angle of water and potassium nitrate solution on the surface of PI and PIS.

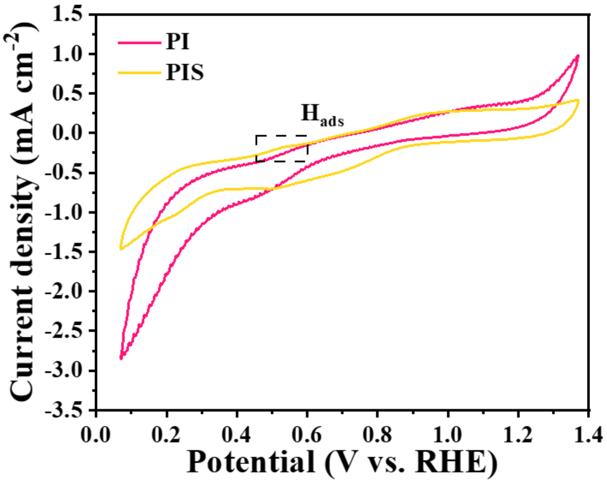

Supplementary Fig. 35. CV curves of PI and PIS in 1.0 M KOH.

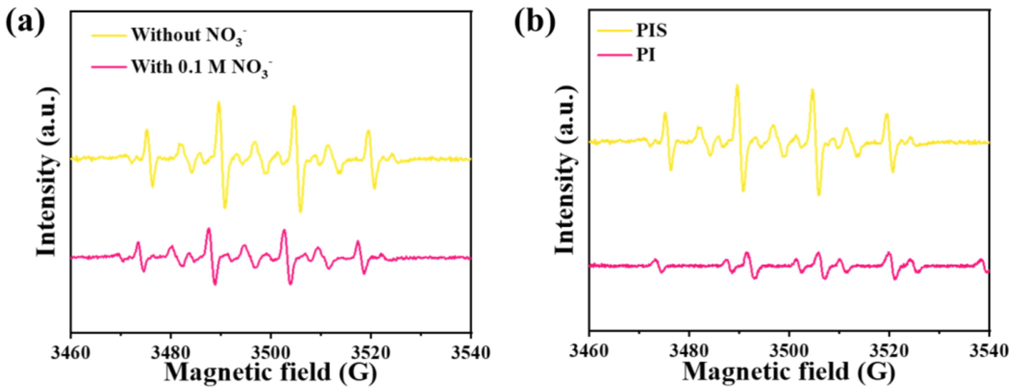

Supplementary Fig. 36. (a) Operando EPR spectra of solutions collected after 10 min of photocatalytic treatment using the PIS system with and without 0.1 M NO_3^-. (b) Operando EPR spectra of solutions collected after 10 min of photocatalytic treatment using PIS and PI systems.

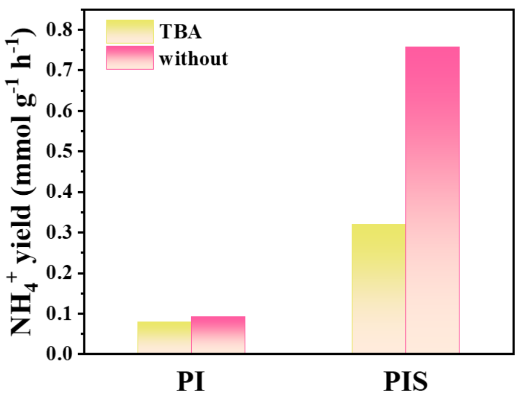

Supplementary Fig. 37. The prepared samples were photocatalytic nitric acid reduction with or without tert-butanol (TBA) (scavenger for the quenching of reactive hydrogen (*H)).

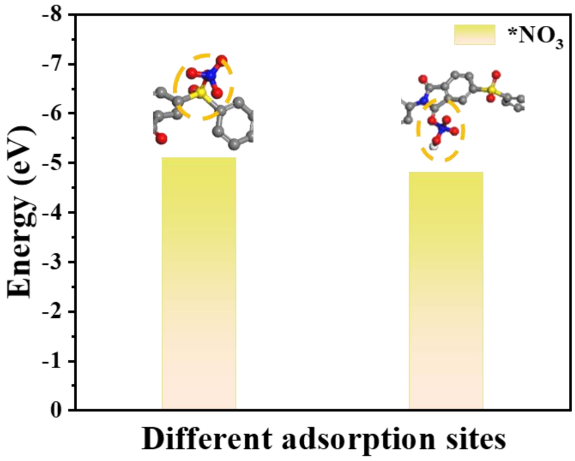

Supplementary Fig. 38. The adsorption energies of *NO_3 at different adsorption sites on PIS.

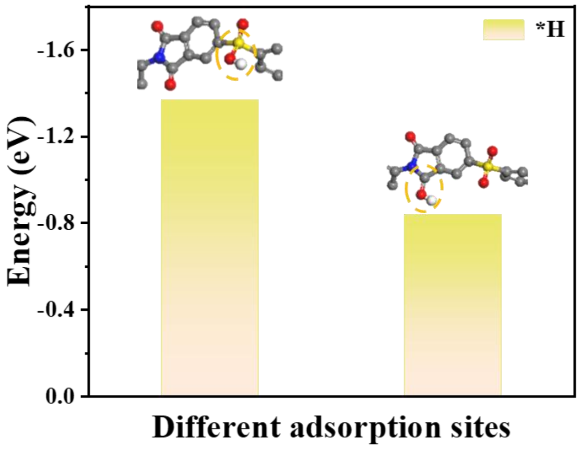

Supplementary Fig. 39. The adsorption energies of *H at different adsorption sites on PIS.

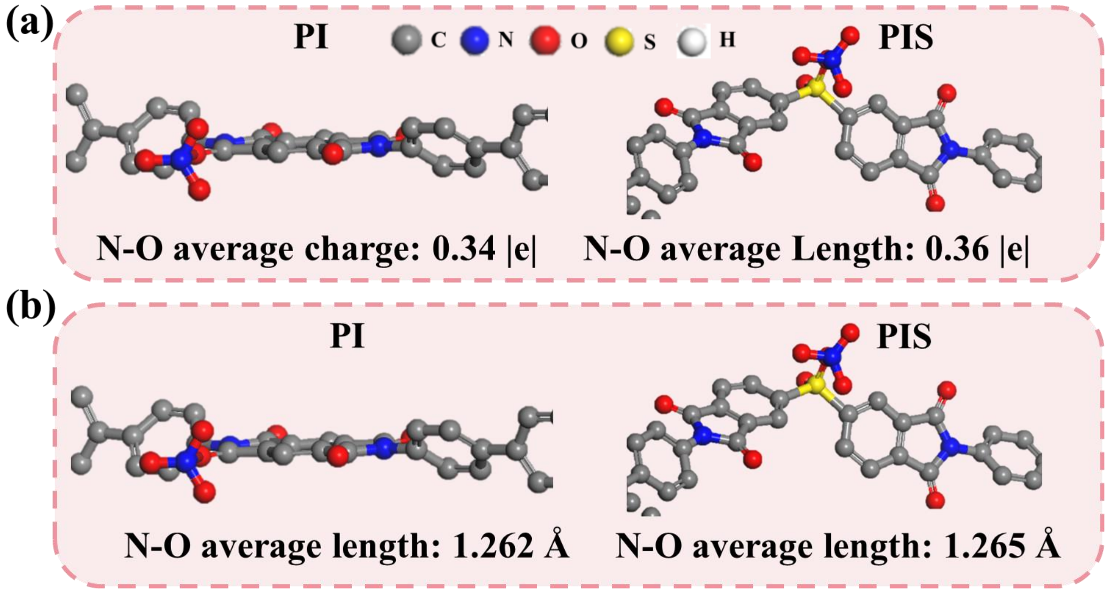

Supplementary Fig. 40. Comparison of (a) average charge density and (b) N-O(3) average bond length between PI-NO_3 and PIS-NO_3.

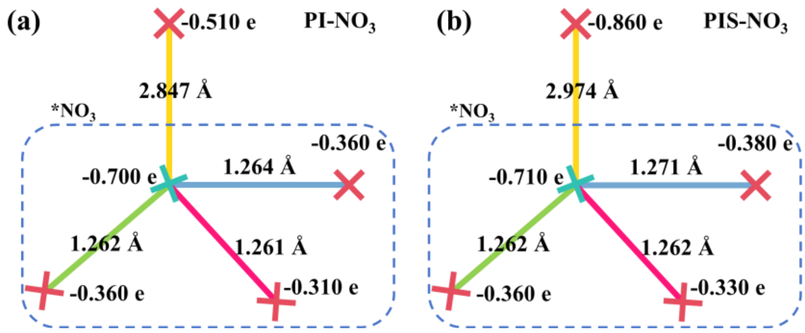

Supplementary Fig. 41. Schematic illustrations of the NO_3 intermediates adsorbed on the surfaces of (a) PI and (b) PIS highlight the differences between the long and short N-O bonds and their corresponding oxygen atomic charges.

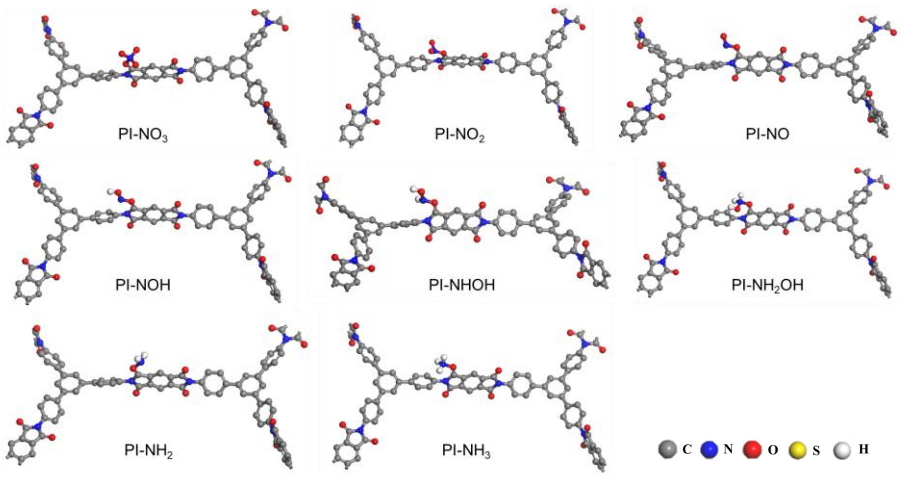

Supplementary Fig. 42. The adsorption reaction flow chart on PI.

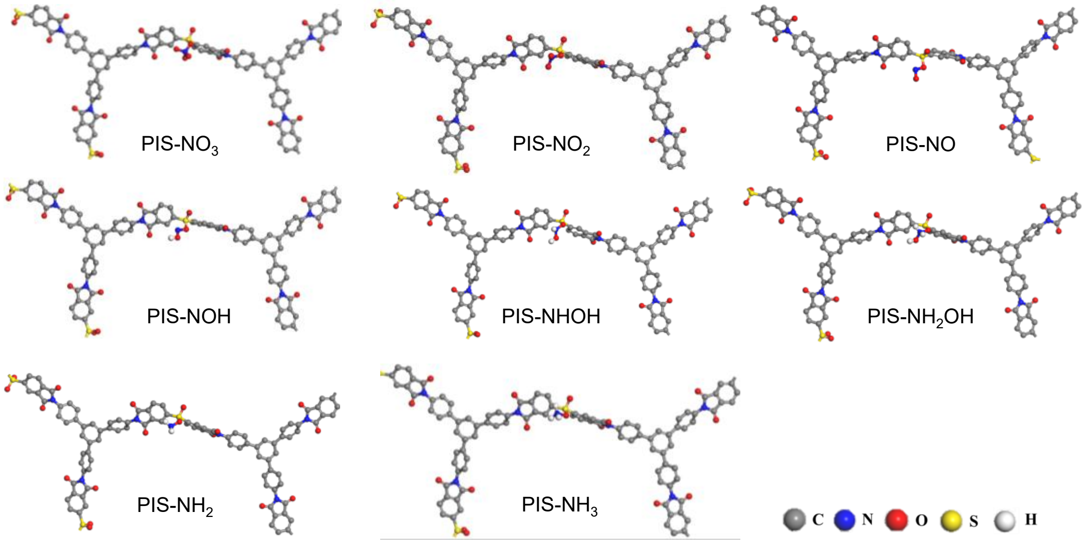

Supplementary Fig. 43. The adsorption reaction flow chart on PIS.

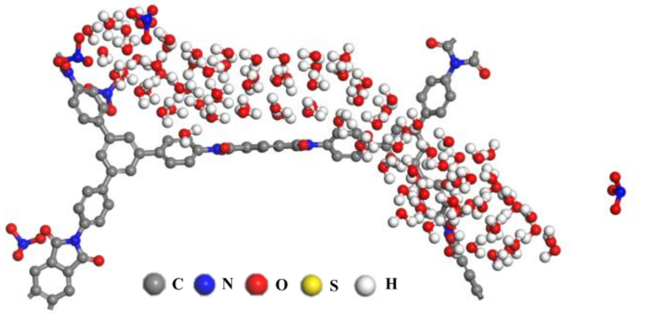

Supplementary Fig. 44. The schematic diagram of water molecule dispersion in PI during molecular dynamics simulation.

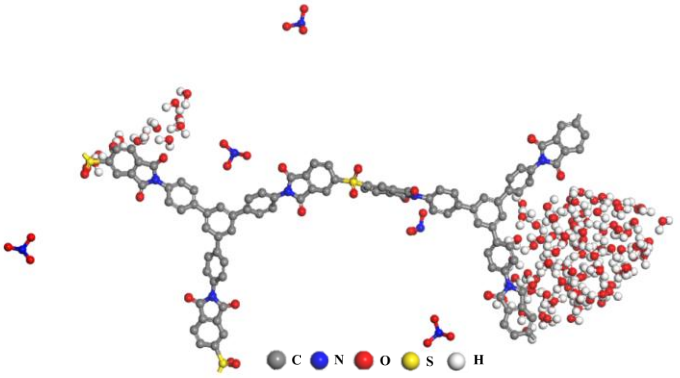

Supplementary Fig. 45. The schematic diagram of water molecule dispersion in PIS during molecular dynamics simulation.

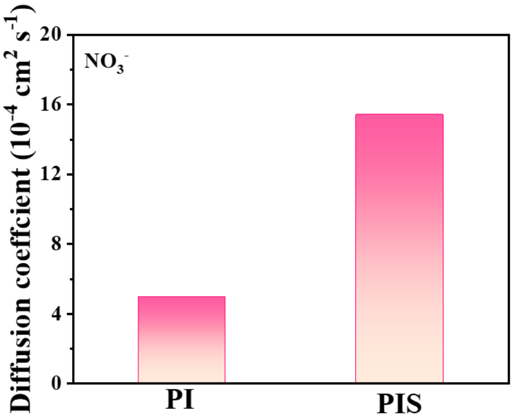

Supplementary Fig. 46. Diffusion coefficients of PIS and PI in nitrate solution.

Reference

1. Zhang J, *et al.* Synergistically flexible-robust effects mediate the dynamic reconfiguration of perylene diimide polymer to enhance piezo-photocatalytic nitrate reduction. *Appl. Catal. B: Environ. Energy* **361**, 124558 (2025).

2. Wang Q, *et al.* Palladium-catalysed C-H glycosylation for synthesis of C-aryl glycosides. *Nat. Catal.* **2**, 793–800 (2019).

3. Kang B, *et al.* Promoting active hydrogen supply for kinetically matched tandem electrocatalytic nitrate reduction to ammonia. *Appl. Catal. B: Environ. Energy* **360**, 124528 (2025).

4. Liu Y, *et al.* Unveiling intrinsic charge transfer dynamics in bone-joint s-scheme heterostructures to promote photocatalytic hydrogen peroxide generation. *ACS Catal.* **14**, 16287–16296 (2024).

5. Yang J, *et al.* A full-spectrum porphyrin-fullerene D-A supramolecular photocatalyst with giant built-in electric field for efficient hydrogen production. *Adv. Mater.* **33**, 2101026 (2021).

6. Jing J, *et al.* Supramolecular zinc porphyrin photocatalyst with strong reduction ability and robust built-in electric field for highly efficient hydrogen production. *Adv. Energy Mater.* **11**, 2101392 (2021).
