# Supplementary material for: Unlocking carrier confluence in covalent organic frameworks for efficient photoreduction of dilute nitrate to ammonia
Source: Nat Commun. 2026 Feb 24;17:3141. doi: 10.1038/s41467-026-69439-4 (PMC13044306; doi:10.1038/s41467-026-69439-4)
Supplement: Supplementary file 2 — Description of Additional Supplementary File [file 41467_2026_69439_MOESM2_ESM.pdf]

Descriptions of Additional Supplementary Files

File name: Supplementary Data 1

Description: Supplementary Data 1 contains the crystallographic information files (CIF) for all materials discussed in the study, along with the CIF files and PDF versions of the kinetic data. The PDF files also include structural diagrams for clear visualization. This dataset supports the structural analysis and kinetic findings presented in the main text.
