# Supplementary figures and images for: Unlocking carrier confluence in covalent organic frameworks for efficient photoreduction of dilute nitrate to ammonia
Source: Nat Commun. 2026 Feb 24;17:3141. doi: 10.1038/s41467-026-69439-4 (PMC13044306; doi:10.1038/s41467-026-69439-4)

**PI-MD:**

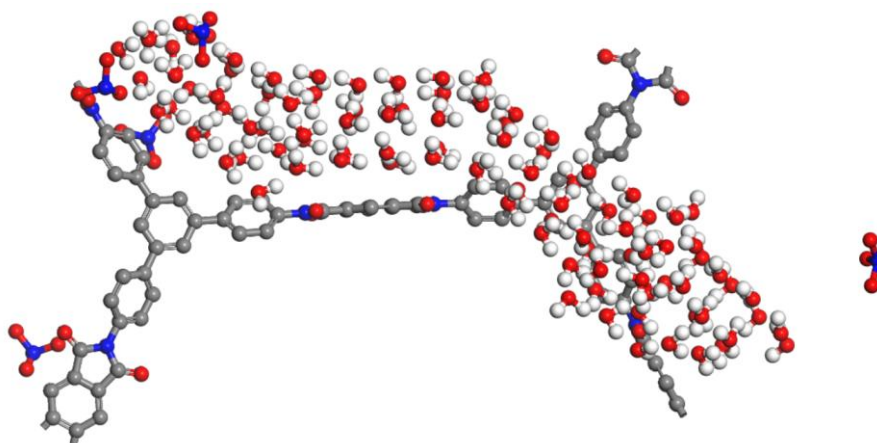

**PIS-MD:**

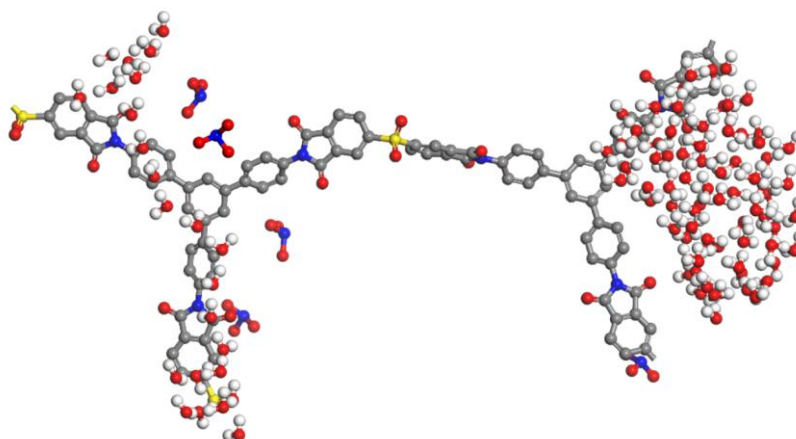

**PI:**

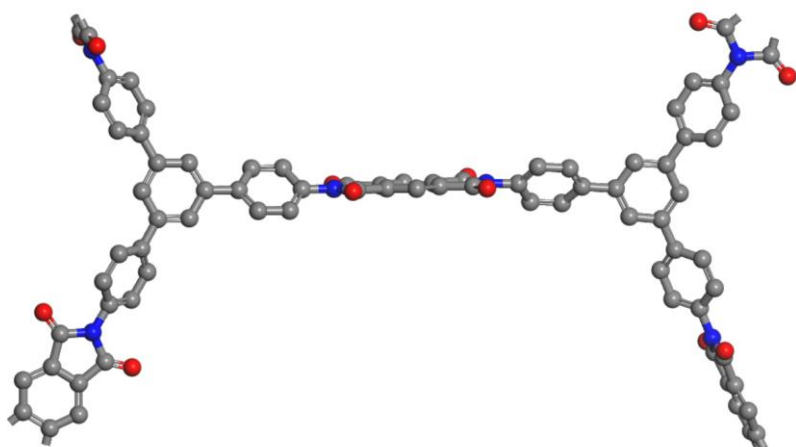

**PIS:**

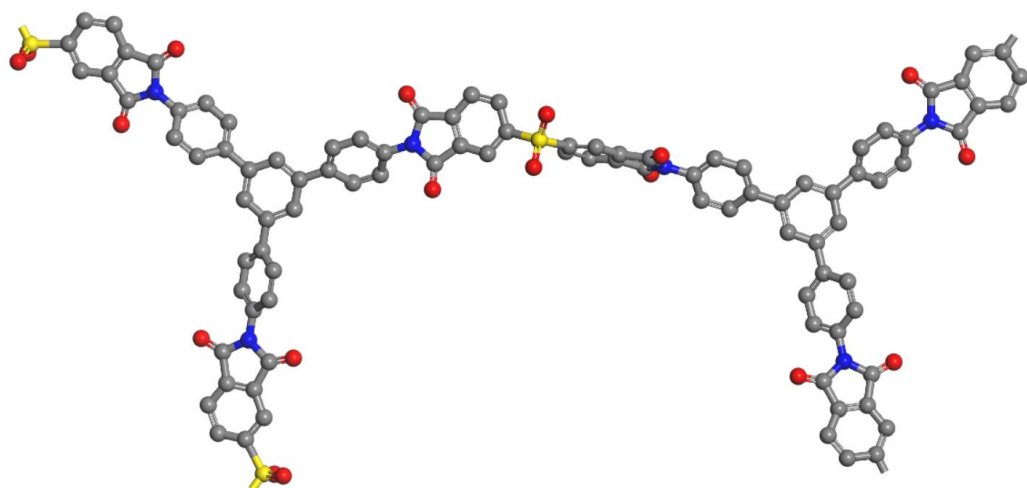

Supplement: Supplementary file 3 — Supplementary Data 1 [file 41467_2026_69439_MOESM3_ESM.zip › Supplementary Data 1/Supplementary Data 1.pdf]
